# A Rare Solitary Fibrous Tumor Localized in the Upper Lip: A Case Report

**DOI:** 10.7759/cureus.83497

**Published:** 2025-05-05

**Authors:** Radvile Raubaite, Sarlota Galinauskaite, Ruta Rasteniene, Linas Zaleckas

**Affiliations:** 1 Institute of Odontology, Faculty of Medicine, Vilnius University, Vilnius, LTU

**Keywords:** cd34, head and neck, sft, solitary fibrous tumor, stat6, upper lip

## Abstract

Solitary fibrous tumor (SFT) is an uncommon mesenchymal neoplasm, rarely localized to the head and neck region. Due to its diverse morphological and histological characteristics, SFT may be misdiagnosed as other soft tissue sarcomas. This case report describes an atypical presentation of SFT involving the upper lip in a 75-year-old man. The only non-specific clinical symptom was localized swelling. An intraoral examination revealed a well-defined, exophytic soft lesion. Magnetic resonance imaging (MRI) revealed a heterogeneous, lobulated lesion with well-defined margins and marked contrast enhancement. The histopathological evaluation demonstrated a proliferation of spindle cells with low mitotic activity within the dense fibrous stroma. Immunohistochemical (IHC) analysis confirmed the diagnosis of benign SFT, showing strong positivity for STAT6, CD34, and Ki-67 markers. Surgical excision of the mass and subsequent histopathological examination of the resected specimen corroborated the initial diagnosis. The patient underwent a follow-up evaluation six months postoperatively, presenting with a fully healed surgical site and no signs of recurrence.

## Introduction

Solitary fibrous tumor (SFT), first described by Klemperer and Coleman, is a rare mesenchymal neoplasm initially identified as fibrous mesothelioma arising from the pleura [1]. Once considered a subtype of mesothelioma or grouped with hemangiopericytoma (HPC) and giant cell angiofibroma, SFT is now recognized as a distinct entity unified by molecular and immunohistochemical (IHC) criteria. The 2021 World Health Organization classification formally retired the term hemangiopericytoma to reflect this updated understanding. Although the precise etiology of SFT remains unknown, its diagnosis is distinguished by a characteristic fusion of the NGFI-A-binding protein 2 (NAB2) and signal transducer and activator of transcription 6 (STAT6) genes, resulting from an intrachromosomal inversion at 12q13, which serves as a key molecular marker, differentiating it from histologically similar neoplasms [2,3]. While epidemiological data are scarce due to evolving definitions, French studies suggest an annual incidence of 3.5 cases per million, accounting for ~5% of soft tissue sarcomas [4]. In the United States, the reported annual incidence of extrameningeal SFT/HPC is approximately one case per two million individuals [5]. Tumors demonstrate an equal prevalence in men and women. Although STF can arise in adolescence, it predominantly affects individuals between 50 and 60, with rare cases reported in infancy [5,6]. As a mesenchymal neoplasm, SFT can arise in various anatomical regions. It is most commonly reported in the thoracic and abdominal region, as well as the retroperitoneal space, whereas occurrences in the head and neck region, particularly the upper lip, are considerably rarer [6]. According to the fifth edition of the WHO classification of Soft Tissue and Bone Tumors, SFT is categorized into benign (locally aggressive), rarely metastasizing, not otherwise specified (NOS), and malignant subtypes. While the prognosis in solitary fibrous tumors is highly variable, risk stratification models incorporating tumor size, mitotic index, and the presence of necrosis assist in classifying cases as benign, locally aggressive, or metastatic. Notably, both benign and malignant variants have the potential to recur or metastasize within the first postoperative year and may, in rare instances, lead to fatal outcomes [6]. The clinical differentiation of SFT is often challenging due to its morphological and histological heterogeneities and potential for malignancy, which may mimic other soft tissue tumors, such as liposarcoma or fibrosarcoma. As a result, an accurate diagnosis requires comprehensive assessment utilizing advanced diagnostic tools. While most SFTs described in the literature are located in the thoracoabdominal region, this report presents the exceptionally uncommon anatomical site of the upper lip, posing unique diagnostic challenges and highlighting the importance of clinical awareness.

## Case presentation

**Figure 1:**
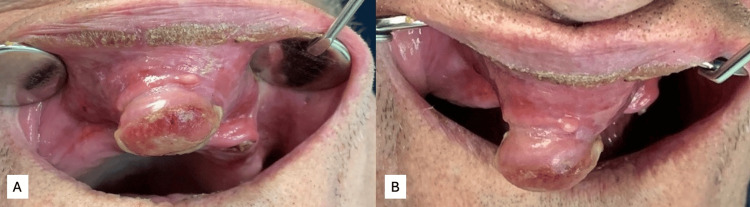
(A, B) Photographs taken during the initial visit showing crusted lips and a large, exophytic, yellowish-pink lesion in the upper lip, prominently protruding into the oral cavity The patient's general oral health is poor, with visible bone atrophy, secondary edentulism, and a remaining upper left canine.

In June 2024, a 75-year-old man presented to the Zalgiris Clinic at Vilnius University Hospital with a localized, painless swelling of the upper lip. According to the patient, the lesion had been progressively enlarging over the past three months, leading to increased discomfort during food mastication, slurring speech, and aesthetic concerns. The patient reported no history of trauma, no genetic illness, and no family history of head and neck tumor-related conditions. However, the patient reported having worked at a nuclear power plant 20 years ago and admitted to smoking up to 20 cigarettes per day. His medical history revealed a prior diagnosis of emphysema and bronchiectasis in 2018, although the treatment was limited to making lifestyle changes, such as quitting smoking and consuming more liquids, which the patient did not comply with. The only prior surgical intervention near the affected area was the extraction of the anterior maxillary teeth; however, the patient was unable to specify the time of the procedure. Clinical examination revealed a yellowish-pink, well-circumscribed, soft, exophytic, painless mucosal lesion. The patient exhibited poor general oral health due to edentulism, except for one remaining tooth stump and crusted lips, signaling possible malnourishment due to mouth incompetence caused by the protruding tumor (Figure 1).

**Figure 2:**
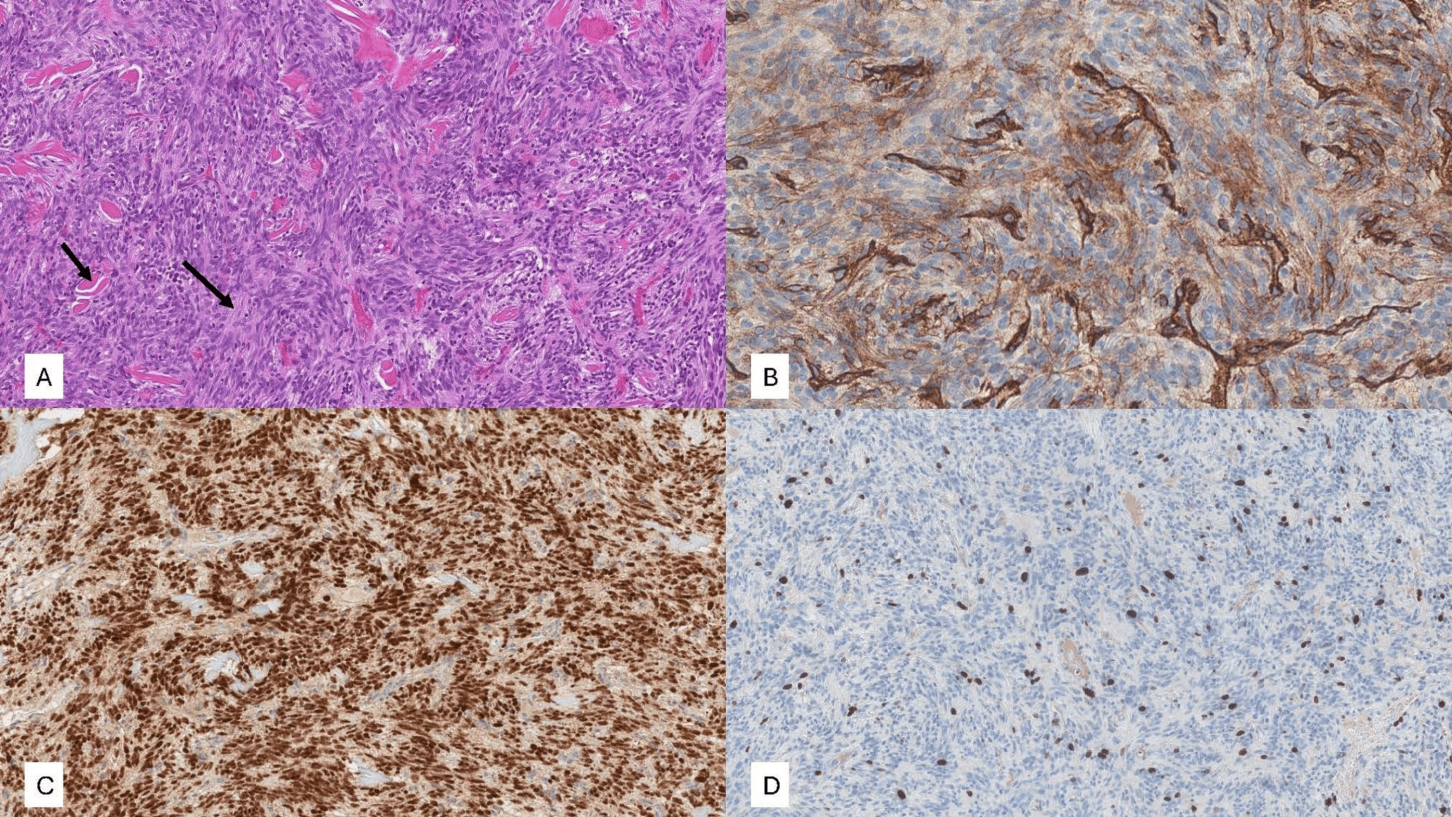
(A) Tumor composed of spindle cells arranged in short fascicles, accompanied by dilated, branching blood vessels and collagen fibers, indicated by black arrows (H&E stain, 10× magnification). (B) STAT6 immunostaining (10× magnification) showing diffuse, strong nuclear positivity in tumor cells. (C) CD34 staining (20× magnification) demonstrating cytoplasmic positivity in tumor cells, with intense brown staining of the vascular endothelium and weaker staining in tumor cells. (D) Ki-67 immunostaining (10× magnification) revealing a proliferative index of approximately 10% in tumor cells

An incisional biopsy was performed for histological evaluation after obtaining written informed consent from the patient. Two mucosal fragments were collected from the upper lip: Fragment 1 (1.0 cm × 0.9 cm × 0.4 cm) and Fragment 2 (0.8 cm × 0.5 cm × 0.6 cm). Macroscopic examination revealed firm mucosal fragments with well-defined borders, without hemorrhagic or necrotic areas on their surfaces. The specimens were processed for histological sectioning, microscopic analysis, and IHC evaluation. Histopathological examination using hematoxylin and eosin (H&E) staining demonstrated a well-defined, non-encapsulated neoplasm composed of spindle-shaped cells (Figure 2). The tumor was analyzed based on fibrous stroma, spindle cell morphology, cellular arrangement, mitotic activity, tumor surface characteristics, and necrosis (Table 1). The IHC analysis was performed using a panel of markers commonly used to identify spindle cell neoplasms (Table 1). The tumor exhibited strong nuclear positivity for STAT6, CD34, and Ki-67 expression. STAT6 nuclear localization is considered a hallmark of SFT, as it is associated with the NAB2-STAT6 gene fusion, a defining genetic alteration specific to this neoplasm.

**Table 1:** Histological and immunohistochemical evaluation of the tumor specimens

Histological examination		Immunohistochemical markers		
Feature	Description	Marker	Reaction	Interpretation
Fibrous stroma	The tumor displayed a dense, fibrous matrix containing collagen fibers and a range of blood vessels of variable size	STAT6	90% nuclear positivity (+++)	Positive (diagnostic for SFT)
		Ki-67	10% nuclear positivity (+++)	Positive (proliferative index)
Spindle cells	The neoplastic cells had a monomorphic appearance, with oval to elongated nuclei and a moderate amount of eosinophilic cytoplasm	CD34	20% cytoplasmic positivity (+/+++)	Positive (consistent with SFT)
Cellular arrangement	The cells were arranged in irregular, interlacing fascicles with a patternless pattern, typical of solitary fibrous tumors	SOX-10	Negative	Rules out nerve sheath tumors
		S-100	Negative	Rules out neural tumors
Mitosis	A low mitotic rate was observed, with a count of one mitosis per 10 high-power fields (HPF)	PanCK	Negative	Rules out epithelial tumors
		HHV8	Negative	Rules out Kaposi's sarcoma
Tumor surface	Surface ulceration was noted, suggesting irritation or local tissue damage	Desmin	Negative	Rules out smooth muscle tumors
		SMA	Negative	Rules out myofibroblastic tumors
Necrosis	No signs of necrosis were present in the tumor	CD68	Negative	Rules out macrophage-like cells
		CD31	Negative	Rules out vascular origin

The tumor was classified according to ICD-10 code D48.7 and morphological codes M8815/0 and M8815/1, corresponding to a benign solitary fibrous tumor.

The prognosis for this case was evaluated using a predictive Demicco model for metastasis risk, incorporating the following factors: patient age (75 years; +1 point for age > 55), mitotic rate (one mitosis per 10 HPF; HPF +1 point), tumor size (<5 cm; 0 points), and absence of necrosis (0 points). A score of 2 points classified the tumor as low risk, while a score of 3-4 points would be considered an intermediate risk, and a score of 5-6 points as high risk for metastasis. The estimated recurrence risk ranges from 10% to 30%, with radical surgical resection recommended as the optimal approach to minimize recurrence likelihood. Given the potential for tumor recurrence or malignant transformation, long-term follow-up is strongly advised to ensure early detection and appropriate management.

**Figure 3:**
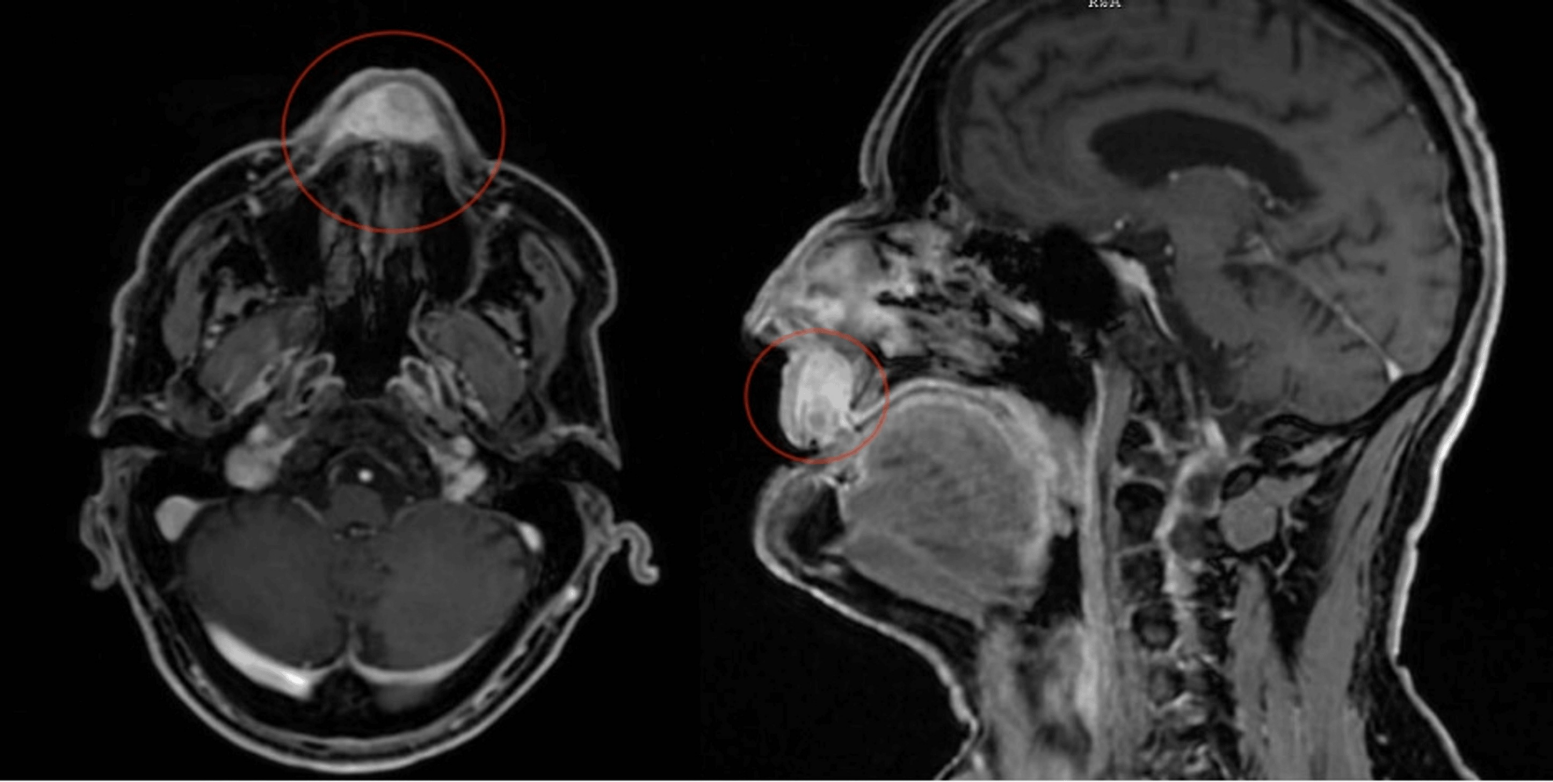
MRI scan (T1-weighted post-contrast sequence) showing the lesion in the surrounding soft tissue of the upper lip Left: axial view. Right: sagittal view. The tumor is indicated by a red circle, with its borders limited to the soft tissues of the upper lip.

An MRI scan evaluated the lesion's size, extent, and characteristics. Imaging revealed a 3.4 cm x 2.6 cm x 1.6 cm mass within the subcutaneous soft tissues of the upper lip, aligned with the midline (Figure 3). The following key radiological features were noted. (1) Well-defined borders: the lesion exhibited clear margins with multilobulated (polycyclic) contours, consistent with a benign, well-demarcated tumor. (2) Heterogeneous lobulated MR structure: the mass displayed variable internal signal intensity, indicating a heterogeneous composition. This characteristic is commonly observed in SFTs due to their fibrous stroma and collagenous matrix. (3) Contrast enhancement: following contrast administration, the lesion demonstrated intense, inhomogeneous enhancement, a feature frequently associated with SFTs, reflecting their vascularity and irregular collagen distribution. (4) Relationship to adjacent structures: the lesion extended toward the lower anterior portion of the nasal cavity but showed no evidence of bone destruction. The absence of bony erosion is a reassuring feature, as malignant or invasive tumors typically demonstrate bone involvement. (5) Neck lymph nodes: no significant cervical lymphadenopathy was detected, and the lymph nodes were considered non-pathological based on MRI findings.

Based on these radiological features, the lesion was considered benign and likely of connective tissue origin. This imaging assessment was consistent with the final diagnosis of a solitary fibrous tumor.

Following preoperative evaluation, the treatment plan, consisting of the surgical tumor excision, follow-up schedule, and full mouth rehabilitation, was discussed with the patient. Tumor observation alone was ruled out due to the lesion’s size and location, the risk of metastasis, and the significant negative impact caused by the tumor on the patient’s quality of life. Given that tumor en bloc excision is the primary treatment modality for SFTs, and considering the potential risks associated with chemotherapy and radiotherapy, such as osteoradionecrosis and medication-related osteonecrosis of the jaw (MRONJ), non-surgical options were ruled out. Postoperative management of mouth rehabilitation proposed to the patient included (1) removal of the remaining upper left canine and fitting of the full removable prosthesis after healing, (2) keeping the canine to maintain bone volume before restoring the maxilla with a fixed prosthesis on the dental implants, and (3) keeping the canine in hopes of restoring its core and using it for prosthesis retention. The patient expressed a desire to preserve the last remaining tooth and make the decision regarding further treatment one year postoperatively, provided there is no recurrence of the tumor.

**Figure 4:**
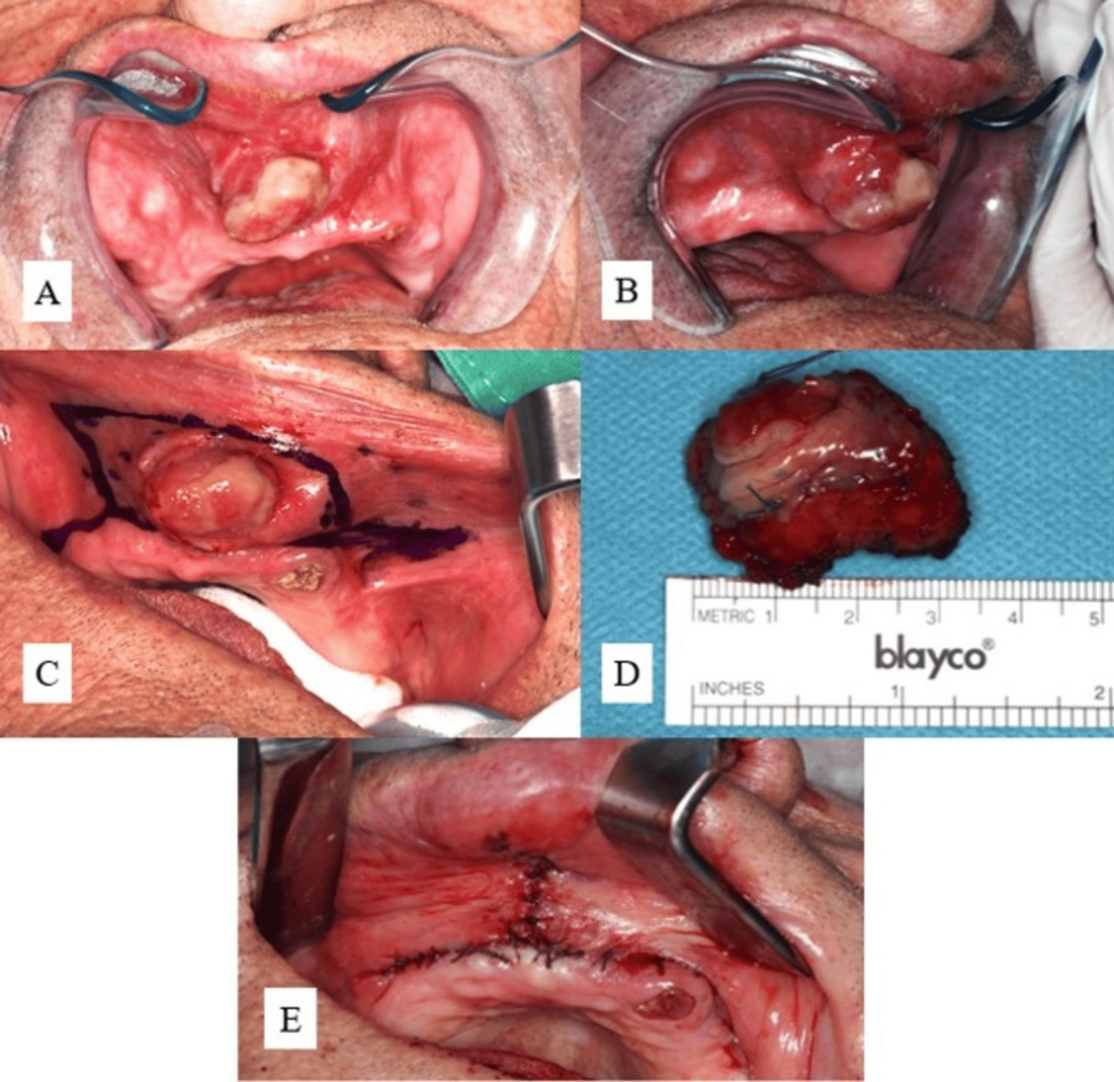
(A, B) Initial exposure of the tumor showing pink-yellowish exophytic lesion of the upper lip. (C) Preoperative marking of the excision accounting for an additional 5 mm of healthy tissues around the tumor. (D) Excised specimen. (E) Closure of the operative wound

Once the treatment plan had been confirmed, the patient was hospitalized for complete tumor resection. The surgical procedure was performed under intravenous sedation; local anesthesia was administered to ensure adequate tumor site anesthesia. An incision was made along the mucosal surface of the upper lip, ensuring adequate tumor-free margins while minimizing disruption to surrounding structures. The lesion’s well-defined borders facilitated its complete en bloc excision. Surgical site closure was performed using absorbable suture material 5/0 Vicryl. The excised specimen, measuring approximately 3.5 x 2.5 x 2.5 cm, was sent for histopathological analysis to confirm the definitive diagnosis (Figure 4).

Postoperatively, the patient received a pharmacological regimen to reduce inflammation, manage pain, and minimize infection risk. The treatment protocol included cefazolin 1 g, intravenous, four times/day; dexamethasone 8 mg, intramuscularly daily; ketorolac 30 mg, intramuscularly daily; and chlorhexidine 0.12 % intraoral rinses following each meal. The postoperative period was unremarkable, and the patient was discharged in stable condition after five days from the hospital.

The secondary pathology report confirmed the diagnosis of SFT involving the mucosa and soft tissues of the upper lip. Excluding the margins of healthy tissues, the tumor itself measured 1.7 x 1.2 x 2.0 cm and was described as a brownish, firm lesion with nodular architecture. Histological examination revealed fibrous and fibromyxoid tissue composition with prominent collagen fibers and variable-sized blood vessels consistent with SFTs' vascular component. 

**Figure 5:**
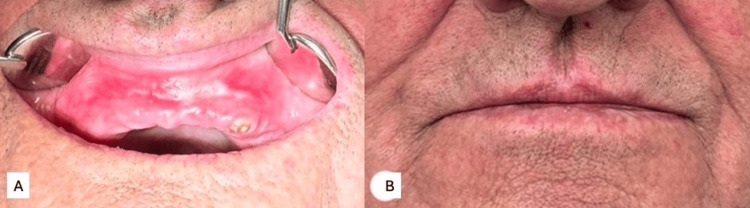
Photographs taken at the postoperative follow-up visit, six months after the surgery (A) Postoperative scarring with otherwise healthy oral mucosa. (B) Taken with the patient's mouth closed, demonstrating adequate healing, restored oral competence, and a favorable aesthetic outcome.

At the six-month follow-up, the patient showed no clinical signs of recurrence. MRI of the primary tumor site and chest computed tomography (CT) were performed to assess local recurrence and distant metastasis. Radiological and clinical findings were unremarkable, with postoperative tissue scarring in the upper lip mucosa as the only residual change. The patient's looks, ability to chew, and oral competence have been restored (Figure 5). The patient reported no complaints and had resumed daily activities. The subsequent follow-up is scheduled for one year postoperatively. In the absence of tumor recurrence, full oral rehabilitation is planned at the patient's primary healthcare center.

## Discussion

SFTs are rare mesenchymal neoplasms that can develop in various anatomical regions, with about 10% occurring in the abdominal cavity, 29% in the extremities, and 22%-29% in the thoracic cavity and pleura [7,8]. The head and neck region, including the oral cavity, is less commonly involved, and lip SFTs are particularly rare. A review of the literature identified only nine documented cases of lip SFTs, with a single specified occurrence in the upper lip of a one-year-old child [9]. Findings underline the rarity of these tumors in the oral region.

SFTs typically exhibit slow, asymptomatic growth, with an average size of 7.5-9 cm, although they tend to be smaller in the head and neck, averaging around 4 cm [6-8]. As the tumor enlarges, functional impairments may arise due to pressure on surrounding structures. For example, sinonasal SFTs may cause nasal obstruction, while laryngeal SFTs are associated with dysphonia in 87% of cases [6]. In the oral cavity, breastfeeding difficulties have been reported in some cases [9]. Our patient presented with an enlarged lesion, which caused slurring speech, difficulty chewing food, and aesthetic concerns; however, other functional disturbances were not observed.

Systemic conditions associated with SFTs include hypertrophic osteoarthropathy, caused by excessive secretion of platelet-derived growth factor (PDGF) and vascular endothelium-derived growth factor (VEDGF), leading to periostitis, arthralgia, digital clubbing, and nail thickening. However, these manifestations were not observed in the current patient [10]. Another paraneoplastic syndrome linked to SFT is Doege-Potter syndrome, which induces hypoglycemia due to excessive secretion of insulin-like growth factor II (IGF2). This occurs in around 7% of SFT cases, although it was ruled out in the present case, as the patient's glucose levels remained normal [10,11].

Radiological imaging plays a crucial role in assessing SFTs, although it is not specific for diagnosis. CT scans often reveal a well-defined, hyperdense mass with contrast enhancement in more than half of cases, indicating significant vascularity [10]. Functional imaging, such as F-18 fludeoxyglucose positron emission tomography (FDG PET-CT), helps assess metabolic activity and heterogeneity [12]. Given the tumor’s size and localization, MRI was the preferred modality in this case. MRI is valuable for detecting hemorrhage, necrosis, and cystic degeneration. In T1-weighted images, SFTs are isointense, while T2-weighted images show variable signal intensities [10]. In the current case, MRI was essential for evaluating tumor borders, its relationship with adjacent structures, and the status of regional lymph nodes.

Macroscopically, SFTs typically present as well-circumscribed, pale whitish-yellow masses [13]. On serosal surfaces such as the buccal mucosa or floor of the mouth, they can appear as exophytic lesions [9]. Microscopically, SFTs consist of spindle-shaped cells within a collagenous stroma, often with characteristic staghorn-like vasculature. They are typically unencapsulated, although the presence of a fibrous capsule remains controversial [13]. Histologically, SFTs share features with other soft tissue neoplasms, necessitating IHC or genetic analysis for a definitive diagnosis. STAT6 and CD34 are commonly used IHC markers. STAT6 has excellent sensitivity, with 100% positivity in most SFT cases. However, it is not entirely specific, as other tumors like liposarcoma and dermatofibrosarcoma can also test positive [3,14]. Similarly, the CD34 marker shows high positivity in 85%-95% of SFT cases, but its diagnostic specificity is limited due to its expression in other neoplasms, including lipomas, myofibromas, and angiofibromas [15]. The decision to proceed with molecular genetic testing strongly depends on the clarity and accuracy of IHC analysis results. For instance, if immunohistochemistry fails to distinguish an SFT from a liposarcoma, molecular genetic testing would reveal a tumor-specific alteration, amplification of the MDM2 gene, a hallmark feature of liposarcoma [15]. However, in our SFT case, the IHC profile (Table 1) was sufficient to exclude similar tumors of fibroblastic, vascular, epithelial, or muscular origin; therefore, genetic testing was not necessary. In addition to STAT6 and CD34 positivities, our case also demonstrated Ki-67 expression. Elevated Ki-67 levels are associated with an increased risk of metastasis within five years post-surgery [16], as seen in this case, where the Ki-67 index was 10%, indicating high cellular proliferation.

Surgical excision remains the primary treatment for SFTs, as inoperable tumors significantly increase the risk of mortality [8]. Additionally, preoperative angiography and embolization are recommended to reduce intraoperative bleeding, especially for highly vascular tumors [10]. In our case, given the tumor’s location and size, electrocautery was deemed sufficient for intraoperative hemostasis. As a result, additional coagulation methods were not employed. 

Perioperative radiotherapy in localized SFTs has been investigated in several retrospective studies. A large retrospective study of 549 patients with localized and resectable SFTs demonstrated that perioperative radiotherapy (RT) can reduce local progression, particularly in intermediate- or high-risk tumors as defined by the Demicco risk stratification system [17]. However, in this patient, the potential risks of RT in the oral cavity outweighed its benefits. Adverse effects such as mucositis, fibrosis, salivary gland dysfunction, xerostomia, and osteoradionecrosis are particularly concerning in this anatomical region. Given the complete resection, low-risk histologic features, and the objective of preserving oral structures for future prosthetic rehabilitation, radiotherapy was excluded from the treatment plan.

Chemotherapy plays a limited role in SFTs due to poor response rates and significant toxicity and is typically reserved for unresectable, recurrent, or metastatic diseases. Agents like doxorubicin and ifosfamide show variable effectiveness and may pose more harm than benefit in localized cases [18]. Similarly, targeted therapies (e.g., pazopanib and sunitinib) have shown promise in advanced or refractory SFTs, leveraging the tumor’s vascular profile and NAB2-STAT6 fusion. However, their predictive value, long-term efficacy, and safety remain under study and are not routinely recommended for early-stage disease [18]. In this case, with a localized and fully resectable tumor and no signs of aggressive behavior, surgery alone was considered both effective and minimally morbid.

Regardless of the treatment modality chosen, long-term surveillance is essential in cases of SFT, including those classified as benign, due to the potential for recurrence. A study on late recurrences of SFTs found that both benign and malignant forms could recur, typically at the original tumor site. Distant metastases, although rare, can occur, often in tumors initially diagnosed as benign [19]. In malignant SFTs of the head and neck, metastases occur in 11% of cases, with 40% involving regional lymph nodes. Mortality risk is influenced by tumor size, metastasis, and resectability, with a 10-year cancer-specific mortality rate of 21% for malignant SFTs in the head and neck [8].

The prognosis for SFTs can be assessed using models like the Demicco risk stratification system, which incorporates factors like mitotic count, tumor size, age, and necrosis [20]. Based on the modified Demicco model assessing tumor size, age, and Ki-67 index, the patient scored as intermediate risk, indicating a 74.1% metastasis-free survival rate at five years [16]. The G-score model, which includes mitotic count, necrosis, and gender as prognostic factors, further supports the intermediate-risk classification. In addition, Georgiesh et al. recommend a detailed follow-up schedule: patients at intermediate risk should undergo check-ups every six months during the first five years and annually thereafter [21]. It is advised to perform an MRI or CT of the primary tumor site and chest radiography to detect distant metastases during follow-up [21]. In accordance with these guidelines, we performed an MRI of the primary tumor site and a chest CT six months after the surgery. No recurrence was observed. However, one of the limitations of this study is the short postoperative follow-up period, considering that tumor recurrence may occur even decades later.

## Conclusions

SFTs are rare mesenchymal neoplasms, with lip localization being exceptionally uncommon. Their diverse morphological and histological features present significant challenges in differential diagnosis. IHC staining, particularly the evaluation of STAT6, CD34, and Ki-67 markers, remains the most reliable diagnostic tool for confirming SFT and assessing the risk of metastasis or recurrence. Surgical excision remains the gold standard treatment for benign, resectable SFTs in the head and neck region. Our case highlights the importance of a comprehensive diagnostic approach, individualized management, and a personalized patient follow-up schedule. Future studies should clearly define the localization of head and neck SFT tumors to accurately determine prevalence and incidence rates while also including cases with extended postoperative follow-up periods.
